# Protein Microarrays: A New Tool for the Study of Autoantibodies in Immunodeficiency

**DOI:** 10.3389/fimmu.2015.00138

**Published:** 2015-04-07

**Authors:** Jacob M. Rosenberg, Paul J. Utz

**Affiliations:** ^1^Department of Medicine, Division of Immunology and Rheumatology, Stanford University School of Medicine, Stanford, CA, USA; ^2^Institute for Immunity, Transplantation, and Infection, Stanford University School of Medicine, Stanford, CA, USA

**Keywords:** autoantibodies, primary immunodeficiencies, autoimmunity, protein microarrays, immunodeficiency

## Abstract

Autoimmunity is highly coincident with immunodeficiency. In a small but growing number of primary immunodeficiencies, autoantibodies are diagnostic of a given disease and implicated in disease pathogenesis. In order to improve our understanding of the role of autoantibodies in immunodeficiencies and to discover novel autoantibodies, new proteomic tools are needed. Protein microarrays have the ability to screen for reactivity to hundreds to many thousands of unique autoantigens simultaneously on a single chip using minimal serum input. Here, we review different types of protein microarrays and how they can be useful in framing the study of primary and secondary immunodeficiencies.

## Introduction

Primary immunodeficiency is integrally tied to autoimmunity. A frequent misconception is that the immune system is a one-dimensional balance: that immunodeficiency is a state of immune system under-activation and autoimmunity is a state of over-activation. Although appealing theoretically, the large majority of diseases in either category contradict such a model. In fact, in many immunodeficiencies, autoimmunity is a persistent and dangerous clinical problem (1, 2). Conversely, in many autoimmune diseases, patients are more susceptible to both common and opportunistic infections (3). These clinical observations suggest a complex relationship between autoimmunity and immunodeficiency.

Measurement of circulating autoantibodies has been a critical tool for defining and understanding clinical autoimmune disease. From diagnostic and research perspectives, autoantibodies have the advantage of being highly stable secreted molecules present in blood at high concentrations. Additionally, they are plausible drivers of tissue damage (4). Unlike T cell receptors, the other mechanism by which adaptive immune cells generate specificity, antibodies directly recognize their cognate antigen without requiring antigen processing and presentation. These advantages make antibodies widely used reagents with applications ranging from biomarkers to therapeutics.

Historically, the first clinically relevant autoantibodies were discovered in the mid-20th century following observations of the morphological effects of serum on leukocytes (5, 6). Over the remainder of the 20th century, new techniques emerged which allowed for greater ease of use and specificity. Techniques such as the radiobinding assay, western blot, and enzyme-linked immunosorbent assay (ELISA) allowed for the detection of antibodies against a specific antigen or epitope, rather than against complex mixtures such as cell lysates (Table 1).

**Table 1:** **Historical and current techniques for the detection of autoantibodies**.

Technique	Description	Key examples
Radiobinding assay	Radioactively labeled antigen is incubated with serum, and radioactivity in the antibody-binding fraction is measured	Used in the detection of double-stranded DNA autoantibodies (66) and insulin autoantibodies (67), although currently less frequently used due to the requirement of radioactive reagents
Immunohistochemistry	A cell line or whole tissue is prepared on a slide and incubated with serum. Antibody binding is measured by visualizing fluorescence or enzyme-mediated color change, usually through the use of a secondary antibody conjugated to an enzyme or fluorophore	Used in some anti-nuclear antibody (ANA) testing (68), autoimmune hepatitis testing (69), and in the characterization of anti-citrullinated protein autoantibodies (70, 71)
Enzyme-linked immunosorbent assay (ELISA)	Antigen is coated onto the surface of a multi-well plate and probed with serum. An enzyme-conjugated anti-human secondary antibody is typically used for colorimetric detection	Used in some ANA testing (72), for the sub-classification of anti-neutrophil cytoplasmic antibodies (73), and for vaccination serologies
Bead-based assays	Antigens of interest are covalently coupled to color-barcoded beads. Beads are incubated with serum, washed, and then incubated with a fluorescent secondary antibody. Fluorescence is detected using a specialized flow cytometer	Bead-based technology can also be used clinically for detection of autoantibodies. Recently it has notably been used for the discovery of anti-cytokine autoantibodies (ACAAs) in the disease Autoimmune Polyendocrine Syndrome Type I (APS-1) (51, 52) and immunodeficiency associated with anti-IFN γ ACAAs (50, 74, 75)
Protein microarray	See text of this article	Widely used in identifying autoantibodies in autoimmune diseases (10), and recently used to identify ACAAs against B-cell activating factor (BAFF) in SLE (18) and ACAAs against type I IFNs in patients with RAG insufficiency

Each of these tools, however, required an *a priori* hypothesis or knowledge of the autoantigen. In a way, autoantibody profiling has been limited to single targets in a manner analogous to the limitations of the study of gene expression prior to DNA microarrays and high-throughput sequencing. To allow for the study of autoantibodies from a proteomic perspective, highly multiplexed tools are needed.

Here, we briefly review assays pertinent to the detection and study of autoantibodies. We then describe different types of autoantigen protein microarrays and discuss the advantages and disadvantages of each platform. We also review the literature of autoantibodies in immunodeficiency and discuss the role of protein microarrays in addressing unanswered questions. Lastly, we close with theoretical insights into the autoantibody response from a systems perspective made possible by the study of autoantibodies with microarrays.

## Protein Microarray Technologies

DNA microarrays revolutionized the study of gene expression. The first generation of DNA microarrays was fabricated using a robotic printer to spot cDNA nucleotide features directly onto a planar surface, while some newer technologies use inkjet printing or maskless photolithography processes. In either case, fluorescently labeled cDNAs are incubated and allowed to hybridize to complementary features on the array. Arrays are washed, and feature binding is detected by a laser scanner (7, 8).

The paradigm shifting advantages of DNA microarrays were their highly multiplexed nature and minimal requirements for sample input, which allowed for an unbiased screen for relevant gene expression. The reproducibility and scalability of DNA microarrays also allowed for the creation of the Gene Expression Omnibus, a database repository of all published microarray data as a rich public resource (9).

Soon after the first DNA microarrays, it was demonstrated that protein microarrays could similarly be used for the detection of protein binding molecules, including autoantibodies in the serum of patients with autoimmune disease (10–14). Protein microarrays have been used as powerful tools to sub-classify patients with autoimmune diseases (15, 16), to monitor disease activity (17), and for the discovery of novel autoantibodies (18, 19). Although protein microarrays can be used to detect many types of molecules that bind to the printed features (20), in this review we will focus on protein microarrays for the detection of autoantibodies.

## Protein Microarray Design and Implementation

**Figure 1:**
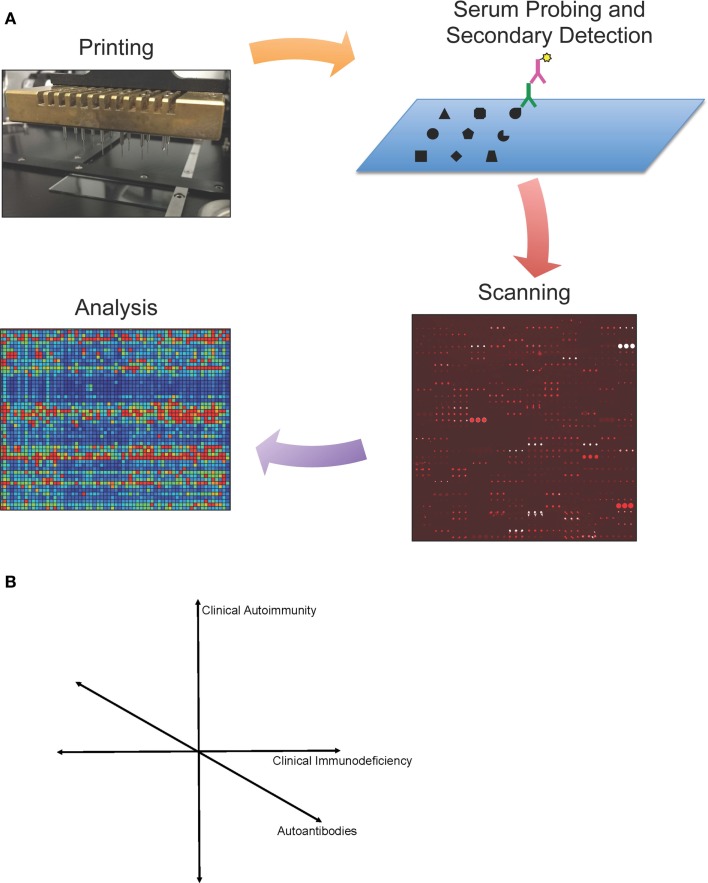
**(A)** Protein microarray technology. Schematic representation of protein microarrays used for autoantibody detection. Antigens are printed onto a specially coated microscope slide surface, and serum antibodies (green) are detected by a fluorescently conjugated secondary antibody (purple). Microarrays are then scanned, and images are analyzed using microarray software. Values are calculated for each antigen based on mean fluorescent intensity and a statistical analysis is performed. Data can be visualized in a heat map representation. **(B)** Simplified schematic representation of proposed map of primary immunodeficiencies.

Protein microarray protocols have been published previously (13, 18, 21, 22). Here, we provide an updated overview of protein microarray processing. We describe our experience and highlight different technologies and approaches relevant to protein microarrays in immunodeficiency. Detection and analysis of autoantibody reactivity by protein microarray have three key steps: (i) array design and fabrication; (ii) array probing, detection, and scanning; and (iii) image processing and data analysis (Figure 1).

### Microarray design and fabrication

Protein microarrays can be designed and fabricated independently or purchased commercially. Array fabrication requires a microarray printer, purified antigens of interest (either expressed in the laboratory or purchased commercially), and a microarray surface on which to print, typically a specially coated microscope slide. Antigens are loaded into one or multiple 384 well plates at either a single concentration or a series of concentrations (our typical protein printing concentration is 200 μg/ml). A typical microarray printer can print on the scale of 100 microarrays over the course of 1 day.

The choice of surface on which to print should be guided by the technical requirements of each laboratory and also the chemistry of the antigens in question. Technically, some microarray scanner detectors are located on the opposite side of the laser source, which precludes the use of opaque microarray surfaces such as nitrocellulose. The two surfaces with which our lab has the most experience are nitrocellulose-coated (Maine Manufacturing) and epoxysilane-coated (SCHOTT) glass slides. The key trade-offs we have observed are that nitrocellulose has high protein-binding capacity, but also a high background fluorescence, which can vary depending on a patient’s serum. Epoxysilane-coated slides have almost no background fluorescence, which has the advantage of not requiring a background fluorescence subtraction step. However, this decreased background comes at the expense of a decreased quantity of protein bound per spot and a corresponding decrease in signal intensity.

Choice of array surface should also take into consideration the nature of antigen printed. Nucleic acids, proteins, carbohydrates, and lipids all have different mechanisms of binding to array substrates. Antigen chemistry should guide array substrate selection, both for adequate attachment of the molecule to the slide surface and to preserve the molecule’s structure. Other surfaces in various stages of development include amine-reactive surfaces, nitrocellulose film coated surfaces (23), plasmonic gold film surfaces for sensitivity enhancement (24), giant magnetoresistive (GMR) biosensor-based surfaces for kinetic measurements (25), and silicon surfaces which allow for high-density photolithographic peptide synthesis (26).

### Antigen content

Over the past decade and a half of protein microarray development, significant progress has been achieved in terms of increasing sensitivity of detection, the number of features printed on each microarray, and the diversity of molecules that can be printed. Initially printed with either nucleic acids or proteins, microarrays have been extended to profile complex mixtures of nucleic acids, proteins, peptides (27), lipids (28–30), and carbohydrates (31–33). Each of these molecules hold their own challenges discussed in the above references.

For laboratories without microarray printing expertise or equipment, protein, peptide, lipid, and carbohydrate microarrays are available from commercial vendors. Commercial microarray products span a spectrum from highly targeted microarrays with high coverage of a specific organism or antigen to broader coverage products with the capability to span the entire known proteome. Examples of targeted products include microarrays from Arrayit onto which key proteins from specific pathogens such as *Plasmodium falciparum* or *Staphylococcus aureus* are printed.

Examples of wide coverage microarray products include the ProtoArray^®^ from Life Technologies, which includes over 9,000 full length human proteins expressed from cDNA constructs in an insect expression system (22). Another example of high-density commercial protein microarrays is peptide microarrays from PEPperPRINT. PEPperPrint sells both prefabricated or custom-printed peptide microarrays fabricated by combinatorial synthesis using laser printing (34).

Such large-scale screens for autoantibodies are appealing in theory, but often the vast number of features also comes at the cost of an increased background signal. Additionally, statistical correction for multiple observations further diminishes signal, in comparison to more focused experiments. Cost can also preclude larger experiments, as prices for each ProtoArray, for example, are approximately $1,000. Lastly, in these high coverage commercial products, each antigen is not necessarily validated beforehand, whereas for custom printed arrays, each antigen can be expressed or purchased and analyzed for quality and activity.

Other high coverage microarrays have been the product of large academic initiatives, most notably from the Swedish groups associated with the Human Protein Atlas initiative (35–37). The Protein Atlas group centered in Stockholm has tightly integrated multiple proteomic technologies including protein expression and monoclonal antibody production, and protein microarrays have been utilized throughout this process. Another center based at Arizona State University has developed nucleic acid programmable protein arrays, in which plasmids are printed on the chip and protein is expressed cell-free, *in situ* (38).

Lastly, there are core facilities, laboratories, and companies which offer printing and processing of microarrays as a send-out service, most notably from the University of Texas Southwestern Genomics and Microarray Core Facility, which offers a full printing, processing, and analysis service (16, 39).

### Microarray probing, detection, and scanning

Probing of microarrays is highly analogous to the probing and detection of a traditional ELISA. Printed microarrays are blocked in a blocking buffer to minimize non-specific binding. Serum is diluted into probing buffer (we typically dilute serum from immunodeficiency patients at 1:100, while for autoimmune diseases with higher signal intensity we dilute at 1:200). Using these dilutions, typically only 1–5 μl of serum or plasma per patient is required. Either serum or plasma can be used; however, it is important to be consistent in the use of either serum or plasma and to not to use them interchangeably, as there are key discrepancies, particularly among low reactivities, between the two blood products.

Given the highly multiplexed nature of the assay, care to avoid confounding factors in processing and analysis is key. Whenever possible, we run samples on the same day and in parallel. Additionally, randomizing the order in which controls and samples are probed aids in minimizing the potential for batch effects, which can be caused by discrepancies in incubation time lengths.

After probing, microarrays are washed and a fluorescent secondary antibody is applied. We typically use an IgG Fc-specific secondary antibody; however, many different isotypes and sub-classes can be measured through the use of isotype-specific secondary antibodies. For experiments where comparisons between isotype abundances are desired, samples can be processed in replicate, or a multi-color detection scheme can be employed (40); we typically use the Cy3 and/or Cy5 fluorescence channels. After incubation with secondary antibodies, the microarrays are then washed and dried. Best results are achieved when microarrays are scanned within 12 h of processing using a fluorescent microarray scanner per manufacturers’ instructions. Care should be taken to avoid repetitive scans of microarrays to avoid photobleaching.

### Image processing and data analysis

High quality microarray images are analyzed using microarray image processing software. The layout and orientation of antigens on the array are stored in one of multiple file formats, and loaded into the image software program, which overlays a corresponding grid of features over the scanned image. The mean fluorescent intensity (MFI) of each individual spot, or “feature,” on the microarray is then exported into a text file. We typically subtract local background fluorescence, and then average replicate features.

Normalization of microarray values should be approached with caution. In ideal settings, we run all samples on the same day to avoid the potential for normalization artifact. When normalization is necessary, median centering is one approach that has been useful (41). However, this approach operates under the assumption that the median value of each patient is equal, a situation that under certain circumstances (e.g., highly polyreactive samples) may not reflect true conditions. In particular, conditions of hypo- or hyper-gammaglobulinemia can alter these distributions. Another approach is to print standard features on each array, such as printing purified IgG or a fluorophore directly onto the array surface, and to normalize to this set of features. However, there can be subtle differences in how features are printed, e.g., the concentration of an antigen can vary due to evaporation during the progression of a print run.

A third approach to normalization when batches are run over multiple days is to use a standard sample with known reactivity as an internal control. Ratios of values from this one sample over different days can then be used as a normalization factor. Taken together, these approaches can be useful normalization tools when necessary; however, normalization should be approached with caution as each of these approaches has its own set of potential confounders.

Post-normalization, statistical differences in autoantibody reactivities can be assessed using significance analysis of microarrays (SAM) (42). SAM is a permutation-based algorithm for significance testing designed for microarray datasets that is less conservative than other corrections for multiple comparisons, such as the Bonferroni correction. In SAM, two or more groups can be compared, although for immunodeficiency studies it is typical to compare one immunodeficiency group to healthy controls. SAM is freely available and can be run either through a script in the R language or using software such as the Multiple Experiment Viewer (43). Through SAM, a false discovery rate can be set and statistically significant autoantibody reactivities identified.

We take the approach that any new result demonstrated by microarray should be replicated on a second independent platform such as ELISA, western blot, or a bead-based assay. This approach reduces the potential for platform-dependent artifacts. Further, platforms like ELISA can be more cost-effective to assess larger cohorts, after using microarray to identify select candidate antigens.

Reproducibility and standardization are critical for any autoantibody assay, and for decades, reference sera have been made available for standardization within and between institutions (44). Given new technologies in monoclonal antibody production, these reference sera can also be replaced with monoclonal antibodies of known affinity and specificity for the antigen of interest. Such monoclonal preparations have the advantage of being in infinite supply. Additionally, given the power of new technologies to discover novel autoantibodies, monoclonal antibodies may be more readily generated as reference standards than identifying reference sera. In our laboratory, we use both monoclonal and serum-based reference standards to ensure reproducibility across tests.

## Current Knowledge of the Autoantibody Landscape in Primary Immunodeficiencies

Given the complex landscape of autoimmunity in immunodeficiency disorders, protein microarrays are a powerful platform to illuminate new aspects of disease pathophysiology and to identify novel biomarkers. Currently, relatively little is known about the full panoply of autoantibodies in primary immunodeficiency.

Most reports have studied individual disease cohorts and evaluated for classical autoantibodies. For example, a recent study found elevated levels of 12 autoantibodies in patients with DOCK8 deficiency (45). Additionally, cohort studies have found autoantibodies in such immunodeficiencies as immune dysregulation, polyendocrinopathy, enteropathy, X-linked (IPEX) (46) and selective IgA deficiency (47). However, larger scale studies of autoantibody reactivities in primary immunodeficiencies are lacking.

We recently profiled autoantibody responses in a blinded cohort of 58 serum samples from healthy individuals and a diverse set of patients with different immunodeficiencies. Using protein microarray data alone, we were able to accurately assign disease based on known anti-cytokine autoantibody reactivities, validating protein microarray technology for broader use. To extend these studies, we profiled a cohort of patients with RAG mutation-associated immunodeficiency and observed the presence of anti-cytokine autoantibodies (ACAAs) against type I interferons.

### Anti-cytokine autoantibodies

A review of proteomics in immunodeficiency would be incomplete without discussion of the burgeoning field of ACAAs, which have been recently expertly reviewed in Ref. (48, 49). Briefly, ACAAs have been found to be tightly correlated with a handful of immunodeficiencies. Perhaps the best described highly sensitive and specific ACAAs include those against interferon γ in atypical mycobacterial infection or other opportunistic infection in Southeast Asia (50). A second key example is ACAAs against type I interferons, which can be observed with or without ACAAs against IL-17A and IL-22, in Autoimmune Polyendocrine Syndrome Type I (APS-1) (51, 52). It is also worth mentioning that the former study was conducted using luciferase-antigen fusion proteins while the latter studies utilized a bead-based system for autoantibody detection. Both of these systems are useful tools with an intermediate level of depth (10–30 antigens) between protein microarrays and individual ELISAs.

Utilizing proteomic approaches to study ACAAs has the potential to be extremely applicable to the study of immunodeficiency on multiple fronts. First, discovery of novel ACAAs has the potential to improve our understanding of disease pathophysiology. Second, proteomic approaches open up new avenues for biomarker discovery. Third, they suggest possible therapeutic opportunities based on cytokine biology. Each of these are active areas of investigation.

Whenever discussing autoantibodies, and in particular with regard to ACAAs, a caveat should be placed that the presence of autoantibodies, even in exquisitely specific patterns, does not demonstrate that those autoantibodies exert a pathologic effect. In fact, there are multiple well-studied autoantibodies that are thought to be markers of disease but do not contribute to the disease process (53). Direct pathologic effect has perhaps been most convincingly shown for the role of ACAAs against GM-CSF in the disease pulmonary alveolar proteinosis, where the cytokine GM-CSF is integrally involved in the disease process, and passive transfer of autoantibodies purified from the serum of patients with the disease reproduces the disease phenotype in non-human primates (54). Yet for the large majority of described ACAAs, to assign a pathologic effect is purely speculative, and more research is needed.

### Autoantibodies in healthy individuals

To understand autoantibodies in any perturbed state such as disease, we first need to understand the basal state in healthy individuals. Unfortunately, little is known about the prevalence and role of autoantibodies in healthy individuals’ immune homeostasis. Clinically it is widely appreciated that rheumatologic disease-“specific” serologies have widely varying levels of specificity (55). The nature and function of autoantibodies in healthy individuals however is poorly understood.

Nowhere is this complexity more apparent than in the study of ACAAs. Multiple studies have found high levels of ACAAs in healthy individuals, including in preparations of intravenous immunoglobulin (56–59). While in some diseases certain ACAAs are specific to a disease, other ACAAs may have variable disease penetrance, acting in combination with other ACAAs or other factors to influence disease. More experiments are needed to answer the questions regarding the prevalence of ACAAs in healthy individuals and whether ACAAs or programs of ACAAs associate with specific genotypes or phenotypes. In particular, large ACAA screens of healthy individuals at different stages of human development and under different natural or experimental perturbations such as vaccination may be highly informative.

## Discussion

Protein microarrays, alongside other proteomic technologies, are valuable tools to define the autoantibody repertoire in primary immunodeficiency. Essential biological questions still remain to be answered. What are the complete autoantibody profiles in each of the primary immunodeficiencies? Are these antibodies pathogenic, and can they be used as biomarkers for screening, diagnosis, or disease monitoring? Additionally, the role of autoantibodies and, in particular, the role of ACAAs in healthy individuals remains to be elucidated.

As we learn more about the autoantibody repertoires in different diseases, we also will have an opportunity to ask systems-level questions. As we generate data on hundreds to thousands of autoantibodies, we can begin to pair these data with other proteomic, genomic, and clinical measurements, hopefully emerging with models of autoimmune regulation across different states of immune function. Given the overlap of autoimmunity and immunodeficiency, these connections may help us better understand both autoimmune and immunodeficiency phenomena.

For example, the immunodeficiency IPEX is caused by mutations in the gene FOXP3. However, there remains a set of “IPEX-like” patients with autoimmunity and enteropathy. We are currently using protein microarrays to profile autoantibody responses in these patients with the hypothesis that sub-groups may have similar prognoses or similar therapeutic response profiles. Another important example is common variable immunodeficiency (CVID), the most common primary immunodeficiency (60). For CVID patients, we have paired autoantibody profiling with genomic and clinical information in order to try to sub-classify this highly heterogeneous disease.

We envision that given sufficient data sets from individual diseases, one can then map primary immunodeficiency diseases in a complex multi-dimensional space. The most intuitive of these approaches would be to map each primary immunodeficiency in a 3-dimensional space of clinical autoimmunity, clinical immunodeficiency, and autoantibodies (Figure 1B). However, each of these axes can be broken down into hundreds or thousands of individual axes, allowing for bioinformatic analysis to compare across diseases. Such analysis could shed new light on the diagnosis, pathophysiology, or treatment of each primary immunodeficiency.

In addition to descriptive profiling studies discussed above, antibody heavy and light chain cloning technologies allow for the expression of monoclonal autoantibodies for use in mechanistic experiments (61–64). Purified monoclonal antibodies allow for *in vitro* and *in vivo* experiments to test the necessity and sufficiency of an antibody for a given phenotype. Once monoclonal antibodies are isolated, protein microarrays can again be used to identify a cognate antigen or antigens.

Lastly, given the growing role of autoantibodies and ACAAs in immunodeficiency, protein microarrays have the potential as diagnostics in the clinic (65). A single microarray chip can screen for several autoantibodies important in both rheumatologic and immunodeficiency disorders. In complex patients with unknown diagnoses, such a chip could fill a significant clinical diagnostic need. In summary, protein microarrays have utility and promise in the field of primary immunodeficiency from basic research through clinical application.

## Methods

The search terms “Autoantibodies and Immunodeficiency” and “Protein Microarray” were queried in the following databases: Pubmed, Scopus, Google Scholar, Stanford University Lane Library, and Web of Science.

## Conflict of Interest Statement

The authors declare that the research was conducted in the absence of any commercial or financial relationships that could be construed as a potential conflict of interest.
